# The activity of SnRK1 is increased in *Phaseolus vulgaris* seeds in response to a reduced nutrient supply

**DOI:** 10.3389/fpls.2014.00196

**Published:** 2014-05-15

**Authors:** Patricia Coello, Eleazar Martínez-Barajas

**Affiliations:** Departamento de Bioquímica, Facultad de Química, Universidad Nacional Autónoma de MéxicoMexico

**Keywords:** nutrient remobilisation, SnRK1, bean seed development

## Abstract

*Phaseolus vulgaris* seeds can grow and develop at the expense of the pod reserves after the fruits have been removed from the plant (Fountain etal., 1989). Because this process involves sensing the reduction of nutrients and the remobilisation of pod reserves, we investigated the effect on sucrose non-fermenting related kinase 1 (SnRK1) activity during this process. Bean fruits removed from the plant at 20 days after flowering (DAF) demonstrated active remobilisation of nutrients from the pod to the seeds. After 5 days, the pod dry weight was reduced by 50%. The process was characterized by a rapid degradation of starch, with the greatest decrease observed on day 1 after the fruits were removed. The pod nutrients were insufficient for the needs of all the seeds, and only some seeds continued their development. Those seeds exhibited a transient reduction in sucrose levels on day 1 after the fruits were removed. However, the normal level of sucrose was recovered, and the rate of starch synthesis was identical to that of a seed developed under normal conditions. Removing the fruits from the plant had no effect on the activity of SnRK1 in the pods, whereas in the seeds, the activity was increased by 35%. Simultaneously, a large reduction in seed sucrose levels was observed. The increase in SnRK1 activity was observed in both the cotyledon and embryo axes, but it was higher in the cotyledon. At 20–25 DAF, cotyledons actively accumulate storage materials. It is possible that the increase in SnRK1 activity observed in seeds developed in fruits that have been removed from the plant is part of the mechanism required for nutrient remobilisation under conditions of stress.

## INTRODUCTION

Seed development is a complex and highly resource-demanding process that requires large amounts of C and N. Because of structural restrictions, legume embryos grow in an environment in which O_2_ represents only 0.4% of the atmospheric concentration, resulting in low ATP levels (Rolletschek et al., 2003). The adaptations of becoming green and photosynthetically active provide the O_2_ and energy required to increase biosynthetic fluxes. Early in development (pre-storage phase), embryos contain high levels of glucose (Weber et al., 1995). Later, sucrose becomes more abundant, and the switch from hexoses to sucrose is accompanied by cell differentiation and the synthesis of storage products (Borisjuk et al., 1995). In cotyledons of *Vicia faba*, high glucose concentrations are found in non-differentiated regions, and glucose concentrations are particularly low in mature starch-accumulating regions (Borisjuk et al., 1998). By contrast, starch-accumulating cells contain the highest sucrose concentrations (Borisjuk et al., 2002). ATP distribution is also important in regulating the accumulation of storage products. In *V. faba* cotyledons, protein accumulation occurs in the foremost regions, where ATP is more readily available, and starch synthesis is more active in the internal sections, where ATP levels are lower (Borisjuk et al., 2003). Sucrose non-fermenting related kinase 1 (SnRK1) is also a modulator of abscisic acid (ABA) functions, linking nutrient and/or energy state to ABA-regulated responses, and reduction in SnRK1 activity may cause either loss of ABA function and/or disconnection between metabolic signals and ABA, resulting in the prolonged expression of genes related to cell proliferation (Radchuk et al., 2006).

Variations in environmental factors (drought, high temperature, disease caused by pathogens, etc.) affect photosynthetic activity and may produce a significant reduction in the supply of nutrients required for seed development. Plants use different strategies to cope with conditions of stress, including nutrient remobilisation (Yang et al., 2001a). This process is normally associated with leaf senescence, where most of the available nutrients are transported to developing seeds (Yang et al., 2001b), but materials accumulated in stems and pods are also important for seed development (Schiltz et al., 2005). Seeds of *Phaseolus vulgaris* can continue their development at the expense of pod reserves when the fruits are removed from the plant at 15–25 days after flowering (DAF; Fountain et al., 1989). SnRK1 has been identified as an important component in the mechanism that allows plants to respond to C and energy deficiencies (Schluepmann et al., 2012). SnRK1 kinases function as heterotrimeric complexes composed of one catalytic subunit (α) and two regulatory subunits (β- and γ-type subunits; Polge and Thomas, 2007). These kinases can phosphorylate and inactivate important enzymes, such as 3-hydroxy-3-methylglutaryl-coenzyme A reductase (HMG-CoA) reductase, sucrose phosphate synthase (SPS), and nitrate reductase (NR; Sugden et al., 1999). SnRK1 also phosphorylates trehalose phosphate synthase (TPS5; Harthill et al., 2006), fructose-6-phosphate, 2-kinase/fructose-2,6-bisphosphatase (F2KP; Kulma et al., 2004) and non-phosphorylating glyceraldehyde 3-P dehydrogenase (Piattoni et al., 2011) and promote their association to 14-3-3 proteins. SnRK1 kinase activity is also required for the redox regulation of ADP glucose PPase (Tiessen et al., 2003). In pea cotyledons, SnRK1 coordinates and adjusts the physiological and metabolic demands of growth (Radchuk et al., 2010), and by mediating transcriptional reprograming in the cells, SnRK1 (AKIN10) also helps plants to survive adverse-energy depleting conditions (Baena-González et al., 2007). SnRK1 activity is under complex regulation, it increases in response to the phosphorylation of the catalytic subunit mediated for GRIK kinases (Shen et al., 2009) and is inhibited by trehalose 6-P (T6P), glucose 6-P, and glucose 1-P (Toroser et al., 2000; Zhang et al., 2009; Nunes et al., 2013b). T6P inhibition of SnRK1 is important in young tissue, and the dissociation constant (K_i_) for the SnRK1-T6P complex is calculated to be closer to 4 (μM; Nunes et al., 2013a). In this context, the main objective of this work is to investigate how SnRK1 activity is affected by the reduction in nutrient supply when bean fruits are removed from the plant.

## MATERIALS AND METHODS

### PLANT MATERIAL

Bean seeds (*P. vulgaris* cv V8025) were grown in a greenhouse at 25°C under a 14 h natural light/10 h dark regime in 3 L plastic pots with agrolite. They were irrigated daily with 150 mL Hoagland solution (Jones, 1982). Flowers were tagged at anthesis, and fruits were removed from the plant at 20 DAF and incubated at 25°C in darkness. In fruits removed from the plant only seeds that were able to continuing their development were analysed.

### SUGAR DETERMINATION

Pod and seed samples (200 mg) were homogenized in 3 mL 80% ethanol and extracted twice at 80°C for 15 min. The soluble fraction was used to determine glucose, fructose, and sucrose. Starch was measured from the insoluble pellet using procedures previously reported (Bernal et al., 2005). Two fruits from three different plants were analyzed.

### PREPARATION OF PLANT EXTRACTS

Plant material was frozen with liquid nitrogen and soluble protein was extracted at 4°C in homogenisation buffer containing 100 mM Tricine-NaOH (pH 8.0), 5 mM DTT, 0.5 mM EGTA, 0.5 mM EDTA, 10% glycerol, 0.02% Brij 35 and 1 mM benzamidine. Prior to the homogenisation 1 mM PMSF, 1X protease inhibitor cocktail (Sigma, Mexico), phosphatase inhibitors (5 mM sodium fluoride, 2.5 mM β-glycerophosphate and 0.2 mM sodium orthovanadate) and insoluble polyvinylpyrrolidone (2% w/v) were added. The homogenate was transferred to microfuge tubes, and insoluble material was removed by centrifugation (13,000 × *g*) at 4°C for 20 min. The supernatant was desalted using an NAP-5 column (GE Healthcare) that was previously equilibrated with homogenisation buffer. Protein content was determined using Bradford reagent (Sigma, Mexico), and the desalted material was used for the SDS-PAGE (Laemmli, 1970). Proteins were visualized by staining with coomassie blue. Specific antibodies for the SnRK1 catalytic subunit were used for western-blot according to previously reported procedures (Fragoso et al., 2009). The phosphorylation of the catalytic subunit was evaluated with with an anti-phospho-AMPKα (T172) antibody (Cell Signaling). Densitometric analysis was performed using Image Lab software (Bio-Rad), and the blot signal was normalized by the amount of protein detected by coomassie blue staining.

### SnRK1 ASSAY

The SnRK1 activity was assayed following a procedure previously reported (Zhang et al., 2009) in 25 μl in microtiter plate wells at 30°C. Assay medium was 40 mM Hepes-NaOH, pH 7.5, 5 mM MgCl_2_, 200 μM ATP containing 0.337 μCi[γ^33^P]ATP (Perkin Elmer), 200 μM AMARA peptide (AMARAASAAALARRR), 5 mM DTT, 1X protease inhibitor cocktail (Sigma, Mexico) and phosphatase inhibitors (5 mM sodium fluoride, 2.5 mM β-glycerophosphate and 0.2 mM sodium orthovanadate). Assays were started with extract (5 μg protein) and after 6 min, 15 μl was transferred to 4 cm^2^ Whatman P81 phosphocellulose paper, immediately immersed in 1% phosphoric acid, then washed with three 800 ml volumes of 1% phosphoric acid, immersed in acetone, dried, and transferred to liquid scintillation vials.

## RESULTS

**FIGURE 1:**
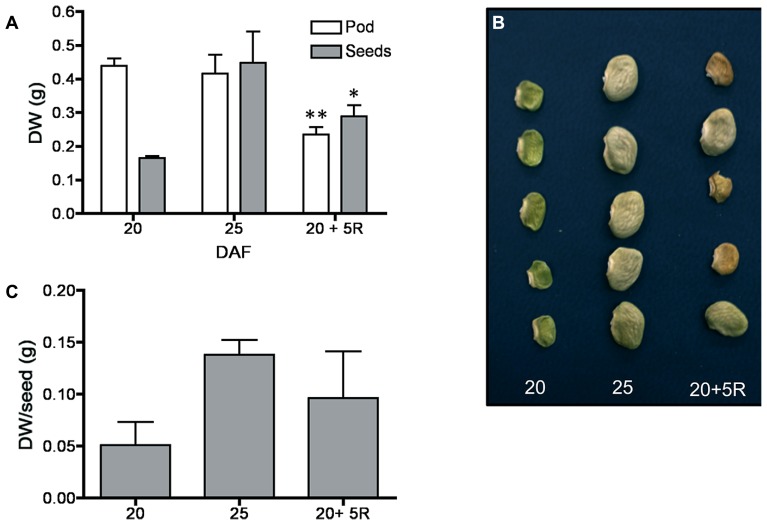
**Transference of pod material to seeds.** Fruits were removed from the plant at 20 DAF; 5 days later (20 + 5R), there was a 50% reduction in pod dry weight and a significant increment in dry weight of seeds **(A)**. Only some seeds in the removed pods continued their development **(B)**, resulting in a large variation in individual seed dry weight **(C)**. Bars represent an average of the analysis of 25 fruits ± SD, * and ** denote significant differences at *p* < 0.05 and 0.01 by ANOVA, respectively. Experiments were replicated three times with identical results.

**FIGURE 2:**
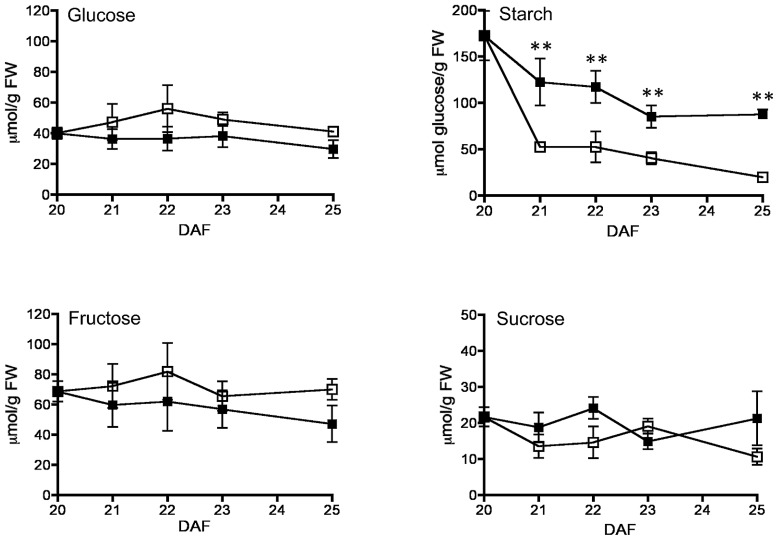
**Effect of fruit removal on pod glucose, fructose, starch, and sucrose levels.** Bean fruits were removed from the plant at 20 DAF (□), and carbohydrate levels were compared to fruits developed attached to the plant (▪). Data points represent the average of three fruits from different plants ± SD, **p < 0.01 by ANOVA. Experiments were replicated three times with identical results.

**FIGURE 3:**
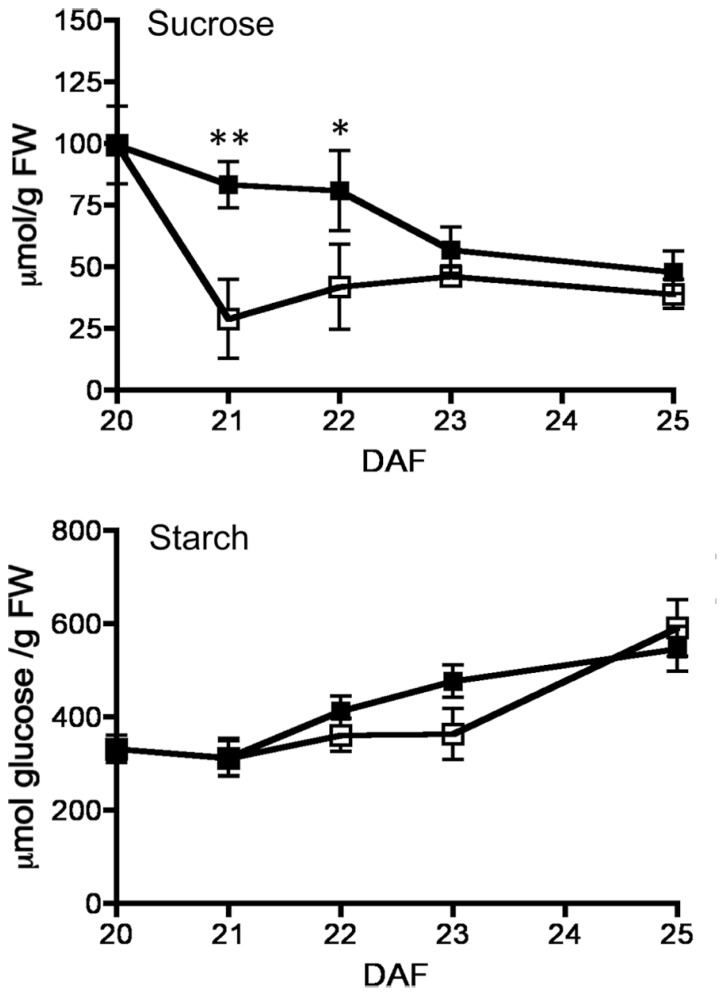
**Effect of fruit removal on the levels of sucrose and starch in seeds.** Bean fruits were removed from the plant at 20 DAF (□), and carbohydrate levels were compared to fruits developed attached to the plant (▪). Data points represent the average of three fruits from different plants ± SD, *p < 0.05 and **p < 0.01 by ANOVA, respectively. Experiments were replicated three times with identical results.

**FIGURE 4:**
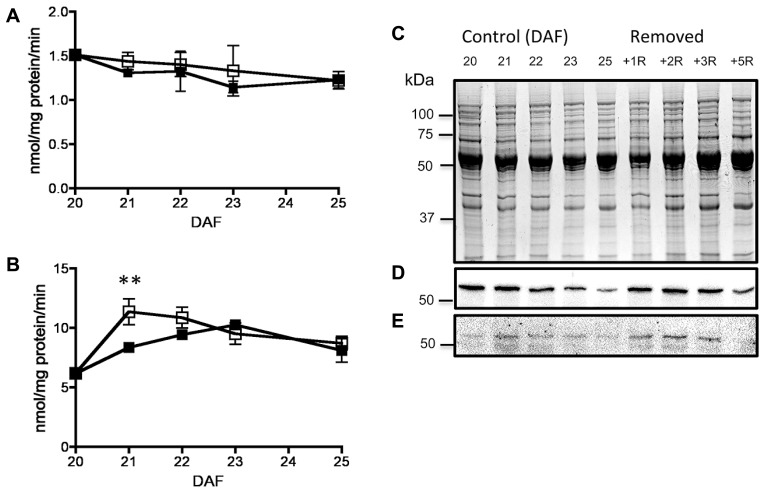
**Effect of fruit removal on the activity of SnRK1 in pods (A) and seeds (B) from fruits removed from the plant at 20 DAF (□) and developed normally (▪).** Data points represent the average of three fruits from different plants ± SD, **p < 0.01 by ANOVA. SDS-PAGE stained with coomassie blue of seed proteins from fruits of 20 to 25 DAF developed under normal conditions (control) or in fruits removed at 20 DAF (removed) and analyzed 1–5 days after **(C)**. Western-blot for total **(D)** and phosphorylated **(E)** SnRK1 catalytic subunit. Experiments were repeated two times with identical results.

**FIGURE 5:**
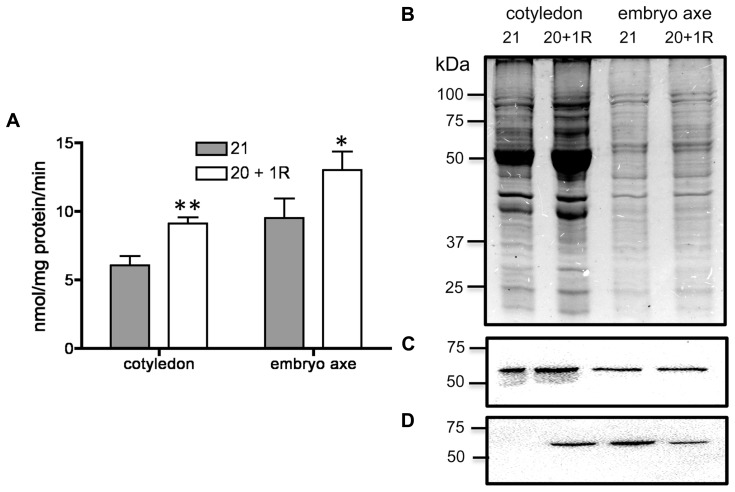
**Analysis of SnRK1 activity in cotyledon and embryo axe from seeds developed under normal conditions for 21 DAF (21) or in fruits removed from the plant at 20 DAF and analyzed at day 1 after (20 + 1R).** Bars represent the average of three independent SnRK1 activity determinations ± SD, *p < 0.05 and **p < 0.01 by ANOVA, respectively **(A)**. SDS-PAGE stained with coomassie blue **(B)** and western-blot for total **(C)** and phosphorylated **(D)** SnRK1 catalytic subunit. Experiments were repeated two times with identical results.

By 20–25 DAF, pods have reached their final size (Fountain et al., 1989), and fruit growth is mainly associated with the active accumulation of storage materials in seeds (**Figure 1A**). In fruits removed from the plant at 20 DAF, seeds can growth at the expense of the nutrients provided for the pod. In the 5 day period after the fruits were detached, the pods lost 50% of their dry weight, whereas a significant dry weight increase was observed in the seeds (**Figure 1A**). However, the material transported from the pod is not sufficient to fully cover the needs of the seeds. **Figure 1B** shows that after 5 days, only some seeds from the removed fruit were able to continue development, and a large dispersion in the size of individual seeds is observed (**Figure 1C**). Bean pods have a significant amount of starch, and approximately one third of it is normally degraded in the period from 20 to 25 DAF. In detached fruits, the process was accelerated, and two-thirds of the starch was hydrolysed during the first day after fruit removal (**Figure 2**). The levels of glucose, fructose and sucrose remained almost constant, and no changes were observed between the pods of fruits developed in the plant and the pods of fruits that were removed (**Figure 2**). Under normal conditions, sucrose tends to be reduced as seed matures. However, in the seeds of fruits that were removed from the plant, an important reduction was observed at day 1. Sucrose levels recovered gradually, and 3 days after the fruits were removed, the levels were identical to those in seeds developed under normal conditions (**Figure 3**). In seeds that were able to continue developing in removed fruits, the rate of starch accumulation decreased during the first 3 days and then returned to values similar to those observed in seeds developed under normal conditions (**Figure 3**). SnRK1 activity in pods showed a tendency to decrease from 20 to 25 DAF, and a similar response was observed in fruits removed from the plant (**Figure 4A**). In seeds, fruit removal resulted in a 35% increase in SnRK1 activity at day 1 (**Figure 4B**). The increase in SnRK1 activity coincided with the largest reduction in sucrose (**Figure 3**). The abundance of total and phosphorylated SnRK1 catalytic subunit was also analyzed. In normally developed seeds, SnRK1 catalytic subunit showed a tendency to decrease after 20 DAF, while in the seeds developed in removed fruits the process is slower (**Figure 4D** and Supplementary Figure 1). In the period from 20 to 25 DAF the phosphorylation of the catalytic subunit is low in seeds developed normally, but it increased at 1–3 days after the fruits were removed from the plant (**Figure 4E** and Supplementary Figure 1). SnRK1 activity was also measured in cotyledons and embryo axes of seeds of 21 DAF and in seeds of 20 DAF fruits the day after removal from the plant. In both cases, removing the fruits from the plant increased the activity with a larger increase observed for cotyledons (**Figure 5A**). The western-blot analysis shows that the increment in SnRK1 activity was not associated with changes in the abundance of catalytic subunit (**Figure 5C**). However, large increment in the phosphorylation of the catalytic subunit was observed in cotyledon extracts, while in embryo axe the phosphorylation of the catalytic subunit is reduced (**Figure 5D**).

## DISCUSSION

At 20 DAF, bean pods have completed their development, and the period from 20 to 25 DAF is characterized by the active growth of the seeds (**Figure 1A**). Later in development, pods can transfer some materials to support seed needs (Schiltz et al., 2005), however, according to previous research (Fountain et al., 1989), when bean fruits are removed from the plant within this period, the pod is converted to provide nutrients for seed development. The present study found that this process is characterized by the rapid reduction of starch, and almost two-thirds of the initially present in pod tissue was degraded during the first day after the fruit was detached (**Figure 2**). In addition to starch, the degradation of other materials may also contribute to seed development. Bean pods also contain galactose-rich pectin polymers that are degraded at the maturation of the fruits (Stolle-Smits et al., 1999), and proteins stored in soybean pods make a significant contribution to the pool of nutrients mobilized for developing seeds (Staswick, 1989). It has been estimated that in *Pisum sativum,* the N remobilised from the pods contributes to 20% of the seed N (Schiltz et al., 2005). By removing the fruits from the plant, both pod and seeds were subjected to a severe nutritional deficiency. This nutrient loss was partially compensated by the acceleration of starch degradation in the pods (**Figure 2**). It is also possible that some seeds are sacrificed to increase the probability to develop some viable seeds (**Figure 1B**). However, the reserves transferred from the pods are insufficient to fully supply the needs of all seeds (**Figure 1**), and a large reduction in sucrose was observed at 1 day after the fruits were detached. Sucrose gradually increased, and after 3 days returned to those levels observed in normally developing seeds, when the rate of starch accumulation was also recovered (**Figure 3**). SnRK1 controls the early steps of cotyledon growth and differentiation and is required to transmit a nutrient-derived signal that stimulates the gene expression involved in nutrient partitioning (Radchuk et al., 2010). SnRK1 also mediates low-energy stress responses (Baena-González and Sheen, 2008). Its activity in the pods was not affected by removing the fruits from the plant (**Figure 4A**). However, in seeds that were able to develop in detached fruits a 35% increase was observed 1 day after its removal (**Figure 4B**). It coincided with the largest reduction in sucrose levels detected in the seeds, and the difference disappeared as soon as the sucrose levels were restored (**Figures 3** and **4B**). Sugar starvation and ABA transcriptionally activates SnRK1 (Radchuk et al., 2010). It might contribute to the higher level of catalytic subunit observed in seeds of detached fruits (**Figure 4D** and Supplementary Figure 1). However, the increment in SnRK1 activity observed in those seeds was associated with larger proportion of the catalytic subunit that appears phosphorylated (**Figure 4E** and Supplementary Figure 1).

In bean seeds, SnRK1 activity reaches its highest point around 20 DAF (Coello and Martínez-Barajas, 2014), and it peaks at 18 DAF in pea seeds (Radchuk et al., 2010). Eventhoug SnRK1 activity is close to the highest value that can be reached in bean seed development, sugar deprivation can produce further increments (**Figure 4B**). It has been estimated that *in vivo*, up to 80% or more of SnRK1 activity is inhibited by T6P (Nunes et al., 2013a). T6P concentration is highly variable, depending on tissue type and environmental conditions (Martinez-Barajas et al., 2011; Nunes et al., 2013a), and generally, T6P levels correlate well with levels of sucrose in plant tissues (Lunn et al., 2006; Martinez-Barajas et al., 2011; Wingler et al., 2012; Nunes et al., 2013a). It has been suggested that the inhibition of SnRK1 activity by T6P allows the cells to initiate the anabolic processes required for growth. When carbon availability decreases, T6P is also reduced, and active SnRK1 participates in the processes required to make carbon available to sink cells into growth (Schluepmann et al., 2012). The highest concentrations of T6P have been reported early in wheat seed development, in which SnRK1 activity is also high (Martinez-Barajas et al., 2011). There is no information available regarding T6P levels in *P. vulgaris* seeds. However, since we did not observed changes in sensitivity of SnRK1 activity to T6P in the seeds of detached fruits (data not shown), we cautiously speculate that a reduction in T6P when sucrose declines (**Figure 3**), will increase the relevance of SnRK1 activity to promote seed development under nutrient deficiency. The data presented here suggest that it is complemented by the changes in the phosphorylation status of the catalytic subunit (**Figure 4E**). The regulation of the phosphorylation of the catalytic subunit could be important to promote differential responses in cotyledon and embryo axe to the nutrient deprivation. Finally, plants provide a number of nutrients to support seed development (sugars, amino acids, minerals, water, among others). On the other hand, SnRK1 is a modulator of ABA functions, linking nutrient and/or energy state to ABA-regulated responses (Finkelstein et al., 2002; Radchuk et al., 2006; Jossier et al., 2009; Rodrigues et al., 2013). It will be important to investigate how the individual nutrients and ABA contribute to the responses observed in seeds of detached fruits.

## AUTHOR CONTRIBUTIONS

Conceived and designed the experiments Patricia Coello and Eleazar Martínez-Barajas. Performed the experiments Eleazar Martínez-Barajas. Analyzed the data Patricia Coello and Eleazar Martínez-Barajas. Wrote the paper Patricia Coello and Eleazar Martínez-Barajas.

## SUPPLEMENTARY MATERIAL

The Supplementary Material for this article can be found online at: http://www.frontiersin.org/Journal/10.3389/fpls.2014.00196/abstract

## Conflict of Interest Statement

The authors declare that the research was conducted in the absence of any commercial or financial relationships that could be construed as a potential conflict of interest.

## References

[B1] Baena-González E. Rolland F. Thecelein J. M. Sheen J. (2007). A central integrator of transcription networks in plant stress and energy signalling. *Nature* 448 938–942 10.1038/nature06069 17671505

[B2] Baena-González E. Sheen J. (2008). Convergent energy and stress signalling. *Trends Plant Sci.* 13 474–482 10.1016/j.tplants.2008.06.006 18701338 PMC3075853

[B3] Bernal L. Coello P. Martinez-Barajas E. (2005). Possible role played by R1 protein in starch accumulation in bean (*Phaseolus vulgaris*) seedlings under phosphate deficiency. *J. Plant Physiol.* 162 970–976 10.1016/j.jplph.2004.12.005 16173458

[B4] Borisjuk L. Rolletschek H. Walenta S. Panitz R. Wobus U. Weber H. (2003). Energy status and its control on embryogenesis of legumes. ATP distribution withing *Vicia faba* embryos is developmentally regulated and correlated with photosynthetic capacity. *Plant J.* 36 318–329 10.1046/j.1365-313X.2003.01879.x 14617089

[B5] Borisjuk L. Walenta S. Roelletschek H. Mueller-Klieser W. Wobus U. Weber H. (2002). Spatial analysis of plant development: sucrose imaging whiting *Vicia faba* cotyledons reveals specific development patterns. *Plant J.* 15 583–591 10.1046/j.1365-313X.1998.00214.x 11846884

[B6] Borisjuk L. Walenta S. Weber H. Mueller-Klieser W. Wobus W. (1998). High-resolution histographical mapping of glucose concentrations in developing cotyledons of *Vicia faba* in relation to mitotic activity and storage process: glucose as a possible developmental trigger. *Plant J.* 15 583–591 10.1046/j.1365-313X.1998.00214.x

[B7] Borisjuk L. Weber H. Panitz R. Manteuffel R. Wobus U. (1995). Embryogenesis of *Vicia faba* L: histodiferentiation in relation to starch and storage protein synthesis. *J. Plant Physiol.* 147 203–218 10.1016/S0176-1617(11)81507-5

[B8] Coello P. Martínez-Barajas E. (2014). SnRK1 is differentially regulated in the cotyledon and embryo axe of bean (Phaseolus vulgaris L) seeds. *Plant Physiol. Biochem.* 80 153–159 10.1016/j.plaphy.2014.03.033 24762788

[B9] Finkelstein R. R. Gampala S. S. Rock C. D. (2002). Abscisic acid signalling in seeds and seedlings. *Plant Cell* 14(Suppl.) S15–S45 10.1105/tpc.010441 12045268 PMC151246

[B10] Fountain D. W. Outred H. A. Holdsworth J. M. Thomas R. G. (1989). Seed development in *Phaseolus vulgaris* L. cv Seminole: I. Developmental Independence of seed maturation. *Plant Physiol.* 89 333–340 10.1104/pp.89.1.333 16666535 PMC1055840

[B11] Fragoso S. Espindola L. Paez-Valencia J. Gamboa A. Camacho Y. Martinez-Barajas E. (2009). SnRK1 isoforms AKIN10 and AKIN11 are differentially regulated in *Arabidopsis* plants under phosphate starvation. *Plant Physiol.* 149 1906–1916 10.1104/pp.108.133298 19211700 PMC2663738

[B12] Harthill J. E. Meek S. E. M. Morrice N. Peggie M. W. Borch J. Wong B. H. C., et al. (2006). Phosphorylation and 14-3-3 binding of *Arabidopsis* trehalose-phosphate synthase 5 in response to 2-deoxyglucose. *Plant J.* 47 211–223 10.1111/j.1365-313X.2006.02780.x 16771775

[B13] Jones J. B. (1982). Hydroponics: it history and use in plant nutrition studies. *J. Plant Nutr.* 5 1003–1030 10.1080/01904168209363035

[B14] Jossier M. Bouly J.-P. Meimoun P. Arjmand A. Lessard P. Hawley S. (2009). SnRK1 (SNF1-related kinase 1) has a central role in sugar and ABA signalling in *Arabidopsis thaliana*. *Plant J.* 59 316–328 10.1111/j.1365-313X.2009.03871.x 19302419

[B15] Kulma A. Vikkadsen D. Campbell D. G. Meek S. E. M. Harthill J. E. Nielsen T. H. (2004). Phosphorylation and 14-3-3 binding of *Arabidopsis* 6-phosphofructo-2-kinase/fructose-2,6-bisphosphatase. *Plant J.* 37 654–667 10.1111/j.1365-313X.2003.01992.x 14871307

[B16] Laemmli U. K. (1970). Cleavage of structural proteins during the assembly of the head bacteriophage T4. *Nature* 227 680–685 10.1038/227680a0 5432063

[B17] Lunn J. E. Feil R. Hendriks J. H. M. Gibon Y. Morcuende R. Osuna D. (2006). Sugar-induced increases in trehalose 6-phosphate are correlated with redox activation of ADPglucose pyrophosphorylase and higher rates of starch in *Arabidopsis thaliana*. *Biochem. J.* 397 139–148 10.1042/BJ20060083 16551270 PMC1479759

[B18] Martinez-Barajas E. Delatte T. Schluepmann H. de Jong G. J. Somsen G. W. Nunes C. (2011). Wheat grain development is characterized by remarkable trehalose 6-phosphate accumulation pregrain filling: tissue distribution and relationship to SNF1-related protein kinase 1 activity. *Plant Physiol.* 156 373–381 10.1104/pp.111.174524 21402798 PMC3091070

[B19] Nunes C. O’Hara L. E. Primavesi L. F. Delatte T. L. Schluepmann H. Somsen G. W. (2013a). The trehalose 6-phosphate/SnRK1 signalling pathway primes growth recovery following relief of sink limitation. *Plant Physiol.* 162 1720–1732 10.1104/pp.113.220657 23735508 PMC3707538

[B20] Nunes C. Primavesi L. F. Patel M. K. Martinez-Barajas E. Powers S. J. Sagar R. (2013b). Inhibition of SnRK1 by metabolites: tissue-dependent effects and cooperative inhibition by glucose 1-phosphate in combination with trehalose 6-phosphate. *Plant Physiol. Biochem.* 63 89–98 10.1016/j.plaphy.2012.11.011 23257075

[B21] Piattoni C. V. Bustos D. M. Guerreo S. A. Iglesias A. A. (2011). Nonphosphorylating glyceraldehyde-3-phosphate dehydrogenase is phosphorylated in wheat endosperm at serine-404 by SNF1-related protein kinase allosterically inhibited by ribose-5-phosphate. *Plant Physiol.* 156 1337–1350 10.1104/pp.111.177261 21546456 PMC3135918

[B22] Polge C. Thomas M. (2007). SNF1/AMPK/SnRK1 kinases, global regulators at the heart of the energy control? *Trends Plant Sci.* 12 20–28 10.1016/j.tplants.2006.11.005 17166759

[B23] Radchuk R. Emery R. J. N. Weier D. Vigeolas H. Geigenberger P. Lunn J. E. (2010). Sucrose non-fermenting kinase 1 (SnRK1) coordinates metabolic and hormonal signals during pea cotyledon growth and differentiation. *Plant J.* 61 324–338 10.1111/j.1365-313X.2009.04057.x 19845880

[B24] Radchuk R. Radchuk V. Weschke W. Borisjuk L. Weber L. (2006). Repressing the expression of the sucrose nonfermenting-1-related protein kinase gene in pea embryo causes pleiotropic defects of maturation similar to an abscisic acid insensitive phenotype. *Plant Physiol.* 140 263–278 10.1104/pp.105.071167 16361518 PMC1326049

[B25] Rodrigues A. Adamo M. Crozet P. Margalha L. Confraria A. Marthino C. (2013). ABI1 and PP2CA phosphatases are negative regulators of Snf1-related protein kinase 1 signaling in *Arabidopsis*. *Plant Cell* 25 3871–3884 10.1105/tpc.113.114066 24179127 PMC3877788

[B26] Rolletschek H. Weber H. Borijuk L. (2003). Energy status and its control on embryogenesis of legumes. Embryo photosynthesis contributes to oxygen supply and is coupled to biosynthetic fluxes. *Plant Physiol.* 132 1196–1206 10.1104/pp.102.017376 12857802 PMC167060

[B27] Schiltz S. Munier-Jolain N. Jeudy C. Burstin J. Salon C. (2005). Dynamics of exogenous nitrogen partitioning and nitrogen remobilization from vegetative organs in pea revealed by 15N in vivo labelling throughout seed filling. *Plant Physiol.* 137 1463–1473 10.1104/pp.104.056713 15793068 PMC1088335

[B28] Schluepmann H. Berke L. Sanchez-Perez G. F. (2012). Metabolic control over growth: a case for trahalose-6-phosphate in plants. *J. Exp. Bot.* 63 3379–3390 10.1093/jxb/err311 22058405

[B29] Shen W. Reyes M. I. Hanley-Bowdoin L. (2009). *Arabidopsis* protein kinases GRIK1 and GRIK2 specifically activate SnRK1 by phosphorylating its activation loop. *Plant Physiol.* 150 996–1005 10.1104/pp.108.132787 19339507 PMC2689985

[B30] Staswick P. E. (1989). Preferential loss of an abundant storage protein from soybean pods during seed development. *Plant Physiol.* 90 1252–1255 10.1104/pp.90.4.1252 16666917 PMC1061877

[B31] Stolle-Smits T. Beekhuizen J. G. Kok M. T. C. Pijnenburg M. Recourt K. Derksen J. (1999). Changes in cell wall polysaccharides of green bean during development. *Plant Physiol.* 121 363–372 10.1104/pp.121.2.363 10517827 PMC59398

[B32] Sugden C. Donaghy P. G. Halford N. G. Hardie D. G. (1999). Two SNF1-related protein kinases from spinach leaf phosphorylate and inactivate 3-hydroxy-3-methylglutaryl-coenzyme A reductase, nitrate reductase, and sucrose phosphate synthase. *Plant Physiol.* 120 257–274 10.1104/pp.120.1.257 10318703 PMC59258

[B33] Tiessen A. Preschaa K. Branscheid A. Palacios N. McKibbin R. Halford N. G. (2003). Evidence that SNF1-related kinase and hexokinase are involved in separated sugar-signalling pathways modulating post-translational redox activation of ADP-glucose pyrophosphorylase in potato tubers. *Plant J.* 35 490–500 10.1046/j.1365-313X.2003.01823.x 12904211

[B34] Toroser D. Plaut Z. Huber S. C. (2000). Regulation of plant SNF1-related protein kinase by glucose-6-phosphate. *Plant Physiol.* 123 403–412 10.1104/pp.123.1.403 10806257 PMC59014

[B35] Weber H. Borisjuk L. Heim L. Buchner P. Wobus U. (1995). Seed-coat associated invertases of Fava bean control both unloading and storage functions: cloning of cDNAs and cell type-specific expression. *Plant Cell* 7 1835–1846 10.1105/tpc.7.11.1835 8535137 PMC161042

[B36] Wingler A. Delatte T. L. O’Hara L. E. Primavesi L. F. Jhurreea D. Paul M. J. (2012). Trehalose 6-phosphate is required for the onset of leaf senescence associated with high carbon availability. *Plant Physiol.* 158 1241–1251 10.1104/pp.111.191908 22247267 PMC3291265

[B37] Yang J. Zhang J. Wang Z. Zhu Q. (2001a). Activities of starch hydrolytic enzymes and sucrose-phosphate synthase in the stems of rice subjected to water stress during grain filling. *J. Exp. Bot.* 52 2169–2179 11604456 10.1093/jexbot/52.364.2169

[B38] Yang J. Zhang J. Wang Z. Zhu Q. Liu L. (2001b). Water deficit-induced senescence and its relationship to the remobilization of pre-stored carbon in wheat during grain filling. *Agron. J.* 93 196–206 10.2134/agronj2001.931196x

[B39] Zhang Y. Primavesi L. F. Jhurreea D. Andralojc P. J. Mitchell R. A. Powers S. J. (2009). Inhibition of SNF1-related protein kinase 1 activity and regulation of metabolic pathways by trehalose 6-phosphate. *Plant Physiol*. 149 1860–1871 10.1104/pp.108.133934 19193861 PMC2663748

