# Supplementary figures and images for: The activity of SnRK1 is increased in Phaseolus vulgaris seeds in response to a reduced nutrient supply
Source: Front Plant Sci. 2014 May 15;5:196. doi: 10.3389/fpls.2014.00196 (PMC4030202; doi:10.3389/fpls.2014.00196)

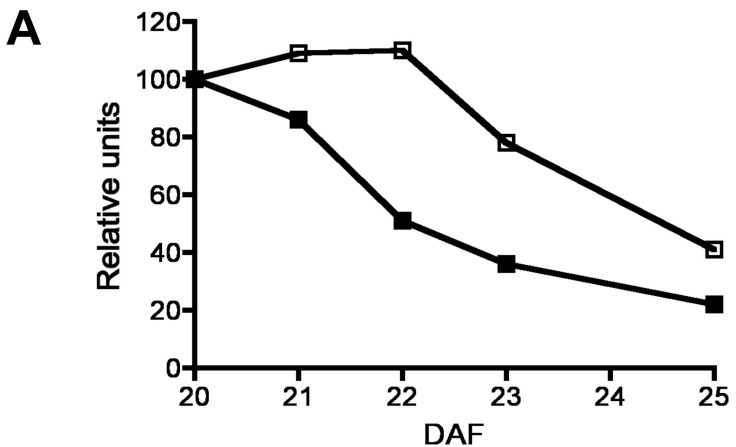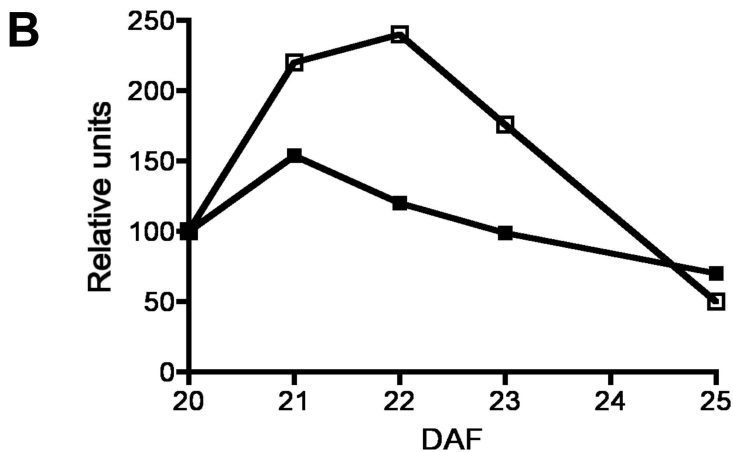

Supplementary figure 1.

Supplement: Supplementary file 1 [file Presentation_1.PDF]
